# Downbeat nystagmus becomes attenuated during walking compared to standing

**DOI:** 10.1007/s00415-022-11106-x

**Published:** 2022-04-12

**Authors:** Haike Dietrich, Cauchy Pradhan, Felix Heidger, Roman Schniepp, Max Wuehr

**Affiliations:** German Center for Vertigo and Balance Disorders, University Hospital, LMU, Munich, Germany

**Keywords:** Downbeat nystagmus, Locomotion, Gaze stabilization, Motor efference copy

## Abstract

Downbeat nystagmus (DBN) is a common form of acquired fixation nystagmus related to vestibulo-cerebellar impairments and associated with impaired vision and postural imbalance. DBN intensity becomes modulated by various factors such as gaze direction, head position, daytime, and resting conditions. Further evidence suggests that locomotion attenuates postural symptoms in DBN. Here, we examined whether walking might analogously influence ocular-motor deficits in DBN. Gaze stabilization mechanisms and nystagmus frequency were examined in 10 patients with DBN and 10 age-matched healthy controls with visual fixation during standing vs. walking on a motorized treadmill. Despite their central ocular-motor deficits, linear and angular gaze stabilization in the vertical plane were functional during walking in DBN patients and comparable to controls. Notably, nystagmus frequency in patients was considerably reduced during walking compared to standing (*p* < 0.001). The frequency of remaining nystagmus during walking was further modulated in a manner that depended on the specific phase of the gait cycle (*p* = 0.015). These attenuating effects on nystagmus intensity during walking suggest that ocular-motor control disturbances are selectively suppressed during locomotion in DBN. This suppression is potentially mediated by locomotor efference copies that have been shown to selectively govern gaze stabilization during stereotyped locomotion in animal models.

## Introduction

Downbeat nystagmus (DBN), a frequent form of acquired fixation nystagmus, is characterized by a spontaneous upward drift of the eyes compensated by fast resetting saccades directed downwards. Patients suffer from visual disturbance due to vertical oscillopsia, to-and-fro vertigo, postural ataxia, and an increased risk of falling [10, 23, 24, 29]. DBN has been associated with impairments in central vestibulo-cerebellar areas [11, 13]. Several hypothetical pathomechanisms have been suggested in the past, such as (1) a central tone imbalance in pathways mediating the vertical vestibulo-ocular reflex (VOR) or smooth pursuit eye movements, or (2) a diminished inhibitory influence of vestibulo-cerebellar Purkinje cells on vertical ocular-motor pathways mediating gaze holding and smooth pursuit [19, 21]. However, the precise etiology of the disease remains hitherto unknown.

The intensity of DBN-related symptoms is known to be modulated by a variety of factors. Accordingly, nystagmus intensity depends on the direction of gaze, the orientation of the head relative to gravity, and illumination [8, 18, 25]. Ocular-motor symptoms typically attenuate during daytime and after prolonged upright resting [26, 27]. More recently it has been found that postural instability in DBN patients is modulated during locomotion: Whereas they exhibit a staggering, broad-based (sensory)-ataxic gait during slow walking, postural deficits diminish during faster walking modes [24]. This locomotion-dependent modulation has been hypothesized to result from a selective suppression of the destabilizing pathological vestibulo-cerebellar influence on balance during fast walking.

It is unknown whether and how locomotion might also affect ocular-motor symptoms in DBN. Symptoms could either improve in a manner analogous to postural stability or even be further aggravated due to the hypothesized central VOR deficits in DBN. Since visual disturbance during walking presents a major risk factor for falling [16], the present study systematically examines gaze stabilizing mechanisms during locomotion as well as walking-related influences on nystagmus intensity in patients with DBN.

## Materials and methods

### Participants

Ten patients with DBN (3 females; mean age 62.6 ± 12.5 years, mean height 172.4 ± 10.4 cm, and mean weight 80.2 ± 17.3 kg) participated in the study (Table 1). Patients underwent a complete neurological and physical examination including testing of sensory and postural dysfunction and an MRI scan of the brainstem and cerebellum. Seven patients had DBN due to an idiopathic etiology and 3 patients had secondary forms of DBN either due to sporadic adult-onset cerebellar atrophy (*N* = 2) or episodic ataxia type 2 (*N* = 1). Ten healthy, age-matched subjects (3 females; mean age 62.8 ± 5.7 years, mean height 173.2 ± 10.8 cm, and mean weight 71.9 ± 7.0 kg) without any auditory, vestibular, neurologic, cardio-vascular or orthopedic disorders served as controls. All participants had normal or corrected-to-normal vision.

**Table 1:** Clinical and nystagmus characteristics of patients

No	Age (y)	Sex	Additional neuro-ophthalmological findings	MRI findings	Etiology	Medication	Time since diagnosis (y)	DBN frequency (Hz)	
								Standing	Walking
1	60	f	1, 2, 3	–	Idiopathic	Fampridine 10 mg 1-0-1	5	2.6	2.3
2	40	m	1, 2, 3, 5	2, 4	Idiopathic	Fampridine 10 mg 1-0-1	12	1.6	0.4
3	58	m	2, 3, 4	1	Idiopathic	Gabapentin 600 mg 1-0-1	2	3.0	2.3
4	45	m	1, 2, 3, 4, 5, 6	–	EA2	4-Aminopyridine 5 mg 1-1-1-1	6	1.8	0.6
5	73	m	1, 2, 3, 4, 6,	1	Idiopathic	Fampridine 10 mg 1-0-1	5	2.2	1.6
6	71	m	1, 2, 3, 5	–	Idiopathic	4-Aminopyridine 5 mg 1-1-1-1	2	2.2	0.9
7	80	m	1, 2, 3, 4, 5	–	Idiopathic	Fampridine 10 mg 1-0-0	2	1.5	1.2
8	71	f	2, 3, 4	2	SAOA	Fampridine 10 mg 1-0-0	2	2.6	1.8
9	63	f	1, 2, 3, 5	2	SAOA	Fampridine 10 mg 1-0-0	1	2.1	0.9
10	65	m	2, 3, 4	–	Idiopathic	Fampridine 10 mg 1-0-0	3	1.1	0.4

Additional neuro-ophthalmological findings: 1: gaze-evoked nystagmus, 2: saccadic smooth pursuit, 3: impaired visual fixation suppression of the vestibulo-ocular reflex, 4: head shaking nystagmus, 5: pathological head impulse test, 6: rebound nystagmus; MRI findings: 1: midline cerebellar atrophy, 2 pancerebellar atrophy, 3 cerebellar neoplasm, 4 cerebellar vascular lesion, 5 cerebellar post-inflammatory lesion; etiology: SAOA: sporadic adult-onset ataxia, EA2: episodic ataxia type 2

### Experimental procedure

Horizontal and vertical eye movements during standing and walking were captured at a sampling rate of 220 Hz using a monocular head-mounted video-oculography system (EyeSeeTec GmbH, Munich, Germany) as described previously [7]. The monocular camera was attached to light-weight goggles, tightly strapped around the subject’s head to prevent slippage. An inertial measurement unit integrated in the center of the camera was used to record 6D head motion (angular and linear). During all recordings, subjects fixated on a red point (visual angle of ~ 0.35°) at a distance of 2.0 m straight ahead of them at standing eye level. For the locomotion experiments, participants walked at their own pace and preferred speed for two minutes on a pressure-sensitive treadmill (Zebris^®^, Isny, Germany; h/p/cosmos^®^, Nussdorf-Traunstein, Germany). DBN patients were secured with a safety belt. Subjects were asked to visually fixate on the red point at all times and to minimize eye blinks during recordings. Recordings during standing and walking were performed in randomized order to control for potential habituation effects.

### Gait analysis

Walking performance of each participant was estimated by calculating the following gait cycle parameters: gait velocity, the mean and the coefficient of variation (CV) of stride length, stride time, base of support as well as the percentage of double support and swing phases with respect to the total gait cycle duration.

### Processing of raw eye movement data

Eye movement recordings were initially screened for potential motion artifacts [7]. Eye blinks during the recordings were identified using a Kalman filter and resulting gaps shorter than 10 ms were closed using cubic spline interpolation in the eye position trace [20]. Larger gaps were excluded from further analysis. Linear head acceleration was integrated to obtain linear head velocity and position. To quantify the impact of linear head motion on image slip, the angular shift of the target that would have been created by the linear motion was calculated using trigonometric functions (see [7] for details). Locomotion recordings were segmented into gait cycles defined by the time points of successive left foot heel contact based on the ground reaction force profiles obtained from the pressure-sensitive treadmill.

### VOR analysis

For the VOR gain calculation, eye velocity was low-pass filtered using a fourth-order Butterworth filter with a cutoff frequency of 10 Hz to remove fast phase eye movements, such as during nystagmus. Angular head velocity and linear head acceleration were filtered analogously. For each locomotion trial, the eye velocity and head angular/linear velocity traces were segmented for successive gait cycles and resampled to the average gait cycle duration of the recording. To quantify VOR responses during walking, the angular and linear VOR gain (slopes of least squares regression of eye *vs* head velocity; see [7]) were calculated for each gait cycle separately and subsequently averaged across all gait cycles.

### DBN frequency analysis

DBN intensity is usually quantified as the mean velocity of the slow upward drift of the eyes (i.e., slow phase velocity). Since during locomotion the DBN-related slow phase eye motions are superimposed by slow, VOR-driven eye movements, it is impossible to determine the exact slow phase velocity of DBN during walking. Thus, to quantify the intensity of DBN symptoms during locomotion, we focused on the mean frequency of DBN quick phases (i.e., the downward-directed resetting saccades) [30]. As a first step, vertical eye velocity was band-pass filtered using a fourth-order Butterworth filter (between 10 and 30 Hz) to remove any low-frequency eye movements (slow VOR responses and DBN-related upward drift) that occur during walking. Fast downward saccades were then identified as DBN quick phases unless they coincided with fast angular (aVOR) or linear (lVOR) VOR responses that occur e.g. during fast head movements after heel strike. These VOR responses were specified as downward eye movements occurring at the same time and with approximately the same magnitude as oppositely directed (upward) head movements. DBN frequency was first quantified as the average frequency of complete standing and walking trials. DBN frequency was further analyzed in relation to the phase of the gait cycle. For the latter, time points of DBN-related quick phases were identified during each gait cycle and normalized to the mean gait cycle duration. DBN frequency was then calculated for 100 equally long time windows throughout the gait cycle. Based on this analysis, the average phase-dependent DBN frequency was computed for each walking trial and a time–frequency density plot of DBN occurrence across the gait cycle was generated from the pooled data of all walking trials of patients (see Fig. 3).

### Statistical analysis

Descriptive statistics are reported as mean ± standard deviation (SD). Differences in gait performance, VOR gains, and nystagmus frequency were analyzed using Student's independent two-sample *t*-test. Activity-dependent differences of nystagmus frequency in patients were analyzed using Student's paired two-sample t-test. Statistical analysis was performed using IBM SPSS (Version 26.0, IBM Corp., Armonk, NY, USA). Results were considered significant at *p* < 0.05.

## Results

### Gait alterations in patients with DBN

Patients and controls showed comparable self-chosen speeds (0.8 ± 0.24 m/s vs. 0.84 ± 0.18 m/s) and step frequencies (107.4 ± 18.8 steps/min vs. 99.0 ± 14.1 steps/min) while walking on the treadmill. Patients exhibited walking alterations typically observed in (sensory)-ataxic gait disorders, with an increased base of support (0.17 ± 0.06 m vs. 0.10 ± 0.03 m, *p* = 0.041) and increased spatiotemporal gait variability (stride length CV: 5.3 ± 2.9% vs. 2.2 ± 0.7%, *p* = 0.005; stride time CV: 4.0 ± 2.6% vs. 1.7 ± 0.6%, *p* = 0.014). Other spatiotemporal gait characteristics were comparable between patients and controls (stride length: 0.89 ± 0.23 m vs. 1.03 ± 0.13 m; stride time: 1.15 ± 0.23 s vs. 1.26 ± 0.19 s, base of support CV: 13.4 ± 8.9% vs. 16.9 ± 6.8%, swing phase: 33.9 ± 2.8% vs. 36.0 ± 2.3%; double support phase: 32.1 ± 5.7% vs. 28.5 ± 4.0%).

### Normal gaze stabilization reflexes during walking in patients with DBN

**Fig. 1:**
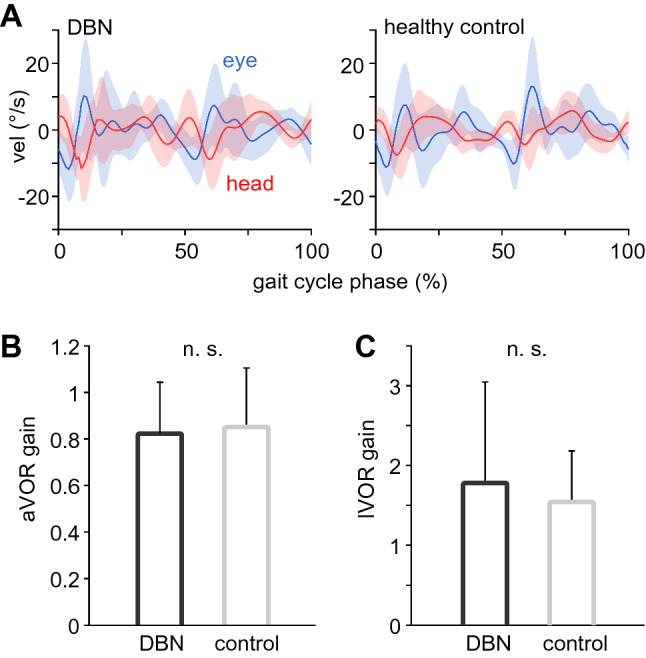
Angular and linear VOR in patients and healthy controls. **A** Mean ± SD vertical angular head (red) vs. eye (black) movements throughout the gait cycle in patients with downbeat nystagmus (DBN; left) and healthy controls (right). **B**, **C** Linear and angular gaze stabilization, i.e., the gain of the angular (aVOR) and linear (lVOR) vestibulo-ocular reflex were comparable between patients and healthy controls

Eye movements need to counteract angular and linear head motion during walking to prevent image slip, especially in the vertical plane. Both vertical eye and angular head movements were comparable between patients and healthy controls (Fig. 1A). Gaze stabilization mechanisms in the angular and linear vertical plane, quantified by the gain of the vertical angular and linear VOR (i.e., aVOR and lVOR), were found to be comparable between patients and healthy controls (Fig. 1B, C). Hence, despite of a continuous nystagmus, eye movements in patients with DBN that are driven by gaze stabilizing reflexes, appear to remain functional.

### DBN frequency decreases during walking

**Fig. 2:**
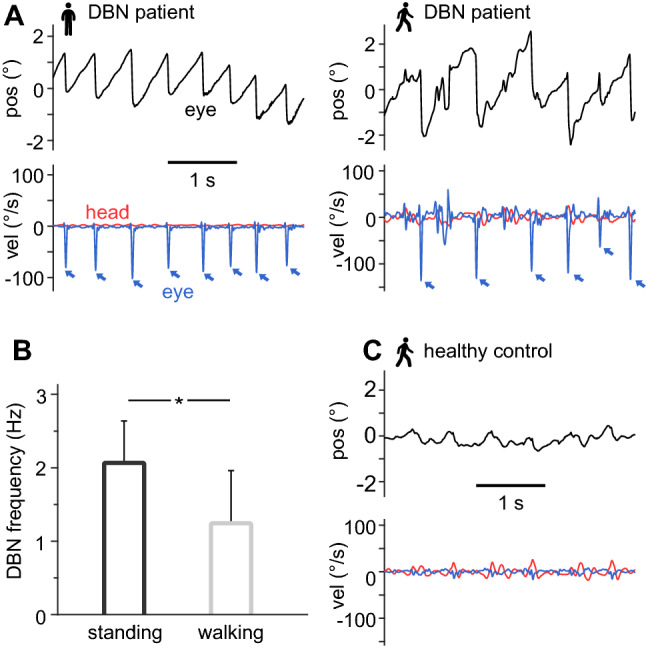
Downbeat nystagmus frequency is reduced during walking. **A** Representative example of eye position (black), eye velocity (blue), and head velocity (red) during standing (left) and walking at preferred speed (right) in a patient with downbeat nystagmus (DBN). Blue arrows indicate occurrence of nystagmus quick phases. **B** Average DBN frequency during standing and walking at preferred speed. **C** Representative example of eye position/velocity and angular head velocity during walking at preferred speed in an age-matched healthy control. * indicates a significant difference

During standing while fixating on a red point straight in front of the head, all patients displayed a continuous upward drift of the eye, compensated by regular downward quick phases (Fig. 2A). DBN frequency ranged between 1.1 and 3.0 Hz (average 2.1 ± 0.6 Hz) (see Table 1, Fig. 2B) in accordance with previous studies [14]. During walking at preferred speed and pace, DBN frequency decreased in all patients to an average value of 1.2 ± 0.7 Hz (*p* < 0.001) (Fig. 2B). In healthy controls, eye movements during walking solely resulted from normal gaze stabilizing mechanisms (i.e., aVOR and lVOR) to compensate concurring head motion (Fig. 1C).

### DBN frequency depends on gait cycle phase

**Fig. 3:**
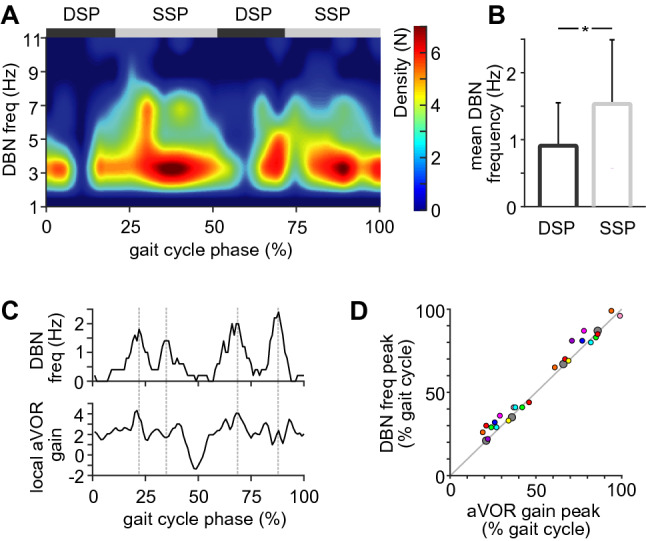
Downbeat nystagmus frequency depends on the gait cycle phase. **A** Density plot of nystagmus occurrence during the gait cycle from the pooled walking trials of all patients. Downbeat nystagmus (DBN) frequency is modulated across the gait cycle and most prevalent during the single support phase (SSP) and the end of the double support phase (DSP). **B** Average differences in DBN frequency between DSP and SSP. **C** Exemplary phase-dependent modulation of DBN frequency (above) and parallel modulation of vertical angular vestibulo-ocular reflex (aVOR) gain across the gait cycle. **D** Peaks of phase-dependent DBN frequency vs. peaks of phase-dependent aVOR gain for all patients closely correspond (large grey dots represent patient data displayed in **C**). * indicates a significant difference

The frequency of DBN was not only affected by walking per se but was further found to be modulated during walking in a manner that depended on the phase of the gait cycle. Time–frequency analysis of nystagmus occurrence across the gait cycle revealed that DBN was more likely to occur during the single support phase (SSP) compared to the double support phase (DSP) of the gait cycle (Fig. 3A–C) (*p* = 0.015). This phase-dependent modulation of DBN frequency resulted in frequency peaks across the gait cycle that closely matched the timing of vertical aVOR gain peaks (see Fig. 3C, D).

## Discussion

Here we studied the impact of locomotion on general gaze stabilizing mechanisms and nystagmus characteristics in patients with DBN. During walking at preferred pace and velocity, patients exhibited a broad-based, staggering stepping pattern compatible with a sensory and/or cerebellar gait disorder [24]. In contrast to postural deficits, their vertical angular and linear gaze stabilization during walking turned out to be fully functional and comparable to that of healthy controls. This agrees with the previous observation of normal and symmetric VOR responses in response to passive head impulses in patients with DBN [9] and contradicts the hypothesis of a central tone imbalance in VOR pathways underlying ocular-motor symptoms in DBN [18, 21].

Spontaneous, downwards directed fixation nystagmus is the primary source for visual disturbance and oscillopsia in DBN. Nystagmus intensity is known to be influenced by static changes in head position relative to gravity [18, 26]. So far it was unknown whether and how DBN might be also influenced by dynamic changes in head orientation such as they occur during locomotion. We observed that walking at preferred pace and speed resulted in a considerable reduction of nystagmus frequency, consistently in all examined patients irrespective of DBN etiology. This attenuation could result from activity-induced changes in head orientation and resultant alterations of vestibular input. DBN has been related to an overacting otolith-ocular reflex [3, 18] according to which the gravity-dependence of DBN should primarily reflect changes in otolithic input [18, 26]. However, due to fixation on a visual target at eye level during walking in our experiment, head orientation of patients was rather kept straight ahead. Moreover, walking has been generally associated with a slight downward tilt of the head in pitch plane [22], which should result in enhancement rather than attenuation of DBN according to the aforementioned assumption [18].

Alternatively, walking-induced attenuation of DBN symptoms could directly arise from locomotor-activity itself. In a previous study we could demonstrate that postural imbalance in DBN becomes attenuated during locomotion in particular during fast stereotyped walking [24]. Analogously, peripheral vestibular ocular-motor impairments such as spontaneous nystagmus in unilateral or deficient VOR in bilateral vestibulopathy are known to improve during locomotion [1, 12]. These observations have been collectively interpreted to reflect a selective central and/or peripheral suppression of sensory feedback during locomotion. A physiological substrate and mechanism for this activity-dependent suppression of sensory feedback has been revealed in amphibian animal models [15, 28]. In these animals, efference copies of the spinal locomotor command are conveyed to upper brain regions where they selectively suppress afferent vestibular input and directly supplement visuo-vestibular reflexes to ensure gaze stability during locomotion. Also in humans, theoretical approaches [4, 17], clinical observations [2, 12], and experimental evidence [5, 6] suggest the presence of an analogous predictive feed-forward regulation of gaze and postural stability during locomotion. Hence, efference copies of the locomotor command could likewise cancel out the destabilizing vestibulo-cerebellar drive that has been suggested to induce central ocular-motor and postural impairments in DBN patients.

A detailed investigation of the timing of remaining nystagmus occurrence during walking further demonstrated that the degree of DBN attenuation during locomotion depends on the specific gait cycle phase. These phasic changes in nystagmus intensity across the gait cycle parallel the phasic modulation of the VOR (Fig. 3C) and vestibulospinal reflexes during human locomotion [5, 6]. Hence, gait cycle phases where DBN is suppressed coincide with those where vertical VOR and vestibulospinal responses are selectively canceled out or attenuated. This observation further suggests that the attenuation of DBN while walking and the suppression of sensorimotor gaze and balance reflexes during locomotion share the abovementioned common mechanism.

In conclusion, the present findings demonstrate that while DBN has profound effects on patients walking ability, it does not impair general gaze stabilizing mechanisms during locomotion. However, walking in turn has an attenuating effect on nystagmus intensity in DBN. This observation parallels previous reports of a locomotion-induced mitigation of sensory ocular-motor and balance deficits during walking. We propose that a common mechanism based on a predictive feed-forward regulation of posture and gaze might explain this attenuation of peripheral and central sensorimotor balance and ocular-motor deficits. Subsequent studies should examine whether, in analogy to postural stability, walking-related attenuation of DBN becomes even more pronounced at non-preferred, fast walking modes. Besides, further investigations are required to explore the functional consequences of the present findings in particular related to the dynamic visual acuity and risk of falling in patients with DBN.
